# Redox Modulation in Hepatic Fibrosis: Translating NOX1/4 Inhibition to Therapy

**DOI:** 10.3390/ijms27010158

**Published:** 2025-12-23

**Authors:** Ghaith K. Mansour, Ahmad W. Hajjar, Irene Marafini, Giovanni Monteleone

**Affiliations:** 1 College of Pharmacy, Alfaisal University, Riyadh P.O. Box 50927, Saudi Arabia; gkmansour@alfaisal.edu; 2 College of Medicine, Alfaisal University, Riyadh P.O. Box 50927, Saudi Arabia; awhajjar@alfaisal.edu; 3 Gastroenterology Unit, Azienda Ospedaliera Policlinico Tor Vergata, 00133 Rome, Italy; marafini@med.uniroma2.it; 4 Department of Systems Medicine, University of “Tor Vergata”, 00133 Rome, Italy

**Keywords:** NADPH oxidase 1 (NOX4), reactive oxygen species (ROS), metabolic dysfunction steatotic-associated liver disease, primary biliary cholangitis

## Abstract

Chronic liver disease (CLD) encompasses a spectrum of progressive disorders, including metabolic dysfunction steatotic-associated liver disease (MASLD) and primary biliary cholangitis (PBC), which together represent a significant global health burden with few effective therapeutic options. The fibrogenic process, common to most forms of CLD, is driven by a complex interplay of cellular stress, inflammation, and wound-healing responses. Nicotinamide adenine dinucleotide phosphate oxidase isoforms 1 and 4 (NOX1 and NOX4) have emerged as key enzymatic sources of reactive oxygen species (ROS), serving as central mediators of hepatic oxidative stress, fibrogenesis, and inflammation. Setanaxib is a first-in-class, orally bioavailable, selective dual inhibitor of NOX1 and NOX4 that has progressed to clinical evaluation. This review synthesizes current knowledge on the molecular pharmacology of the NOX1/4 axis, preclinical evidence from translational models, and clinical trial outcomes to critically assess the therapeutic potential of targeted NOX inhibition in hepatic fibrosis. By attenuating hepatic stellate cell activation, modulating TGF-β signaling, reducing extracellular matrix (ECM) deposition, and regulating hepatic macrophage polarization, setanaxib exhibits pleiotropic antifibrotic effects. The compound also demonstrates favorable pharmacokinetic properties and a good safety profile in patients with PBC, with emerging evidence suggesting meaningful improvements in fatigue and quality of life. Finally, we examine the complex, and sometimes paradoxical, roles of NOX4 in liver pathophysiology, compare the evolving therapeutic landscape with other approaches such as farnesoid X receptor (FXR) agonists, and propose future paradigms integrating artificial intelligence–driven predictive modeling to optimize patient stratification and therapeutic response in this new era of redox-targeted hepatoprotective therapy.

## 1. Introduction

Chronic liver disease (CLD) remains a major global health burden, affecting over one-quarter of the world’s population and contributing substantially to morbidity and mortality [1]. Among the spectrum of CLD, metabolic dysfunction-associated steatotic liver disease (MASLD) has emerged as a leading cause. Regardless of etiology, hepatic fibrosis is the shared pathological outcome, resulting from sustained injury and dysregulated wound-healing processes. The excessive deposition of extracellular matrix (ECM) disrupts liver architecture, progressing to cirrhosis, liver failure, or hepatocellular carcinoma (HCC) [2].

Reactive oxygen species (ROS) are central mediators of fibrogenic signaling. While ROS can originate from multiple cellular sources, the NADPH oxidase (NOX) family (NOX1–5, DUOX1–2) is unique in producing ROS as a regulated enzymatic function rather than as a metabolic byproduct [3,4]. In hepatic cells, NOX1, NOX2, and NOX4 contribute to key pathogenic processes, including hepatic stellate cell (HSC) activation, hepatocyte apoptosis, inflammatory signaling, and ECM remodeling [5].

The direct link between NOX-mediated oxidative signaling and fibrogenesis establishes NOX inhibition as a compelling therapeutic strategy. Despite its promise, a comprehensive and critical evaluation of the evidence supporting the first generation of clinical-stage NOX inhibitors has been lacking. This review aims to fill that gap by systematically assessing the molecular pharmacology, preclinical efficacy, and emerging clinical data for setanaxib, a selective dual NOX1/4 inhibitor. We further delineate its therapeutic potential as a novel antifibrotic agent, with a focus on its development for primary biliary cholangitis (PBC) and its rationale for use in MASLD.

## 2. The NOX1/NOX4 Axis: Distinct Mechanisms in Hepatic Signaling and Fibrosis

NOX1 is primarily localized to the plasma membrane and is an inducible enzyme requiring the assembly of cytosolic regulatory subunits, such as Rac1, for activation. Upon stimulation by agonists like angiotensin II, NOX1 generates superoxide (O_2_^−^), contributing to acute inflammatory responses and cell proliferation [6,7]. In contrast, NOX4 is constitutively active and does not require inducible assembly with cytosolic subunits for its function. It is predominantly localized to mitochondria and other intracellular membranes (Table 1). Uniquely, NOX4 directly produces hydrogen peroxide (H_2_O_2_), a more stable and membrane-permeable reactive oxygen species (ROS), positioning it as a key regulator of intracellular redox signaling [4,8,9,10].

NOX1-derived superoxide is highly reactive and short-lived, driving rapid inflammatory and proliferative signaling. In contrast, NOX4-derived H_2_O_2_ functions as a more classical second messenger, capable of diffusing across membranes to modulate a broader range of redox-sensitive pathways. For instance, H_2_O_2_ can oxidatively inactivate protein tyrosine phosphatases, thereby prolonging growth factor receptor signaling and amplifying profibrotic responses [4,11].

During chronic liver injury, these isoforms play complementary roles. NOX1 is implicated in the initial inflammatory response and HSC proliferation, whereas the constitutive activity of NOX4 is critical for the sustained activation of HSCs into collagen-producing myofibroblasts and for maintaining the chronic fibrogenic state [6]. This dual contribution of NOX1 and NOX4 underscores the rationale for dual inhibition, making agents such as setanaxib particularly attractive as antifibrotic therapies.

## 3. Molecular Mechanisms of NOX1/4-Driven Hepatic Fibrogenesis

The profibrotic effects of NOX1 and NOX4 are mediated through their influence on several core pathological processes, including HSC activation, apoptosis, inflammation, and extracellular matrix (ECM) remodeling [6].

Quiescent HSCs transdifferentiate into proliferative myofibroblasts in response to chronic liver injury, becoming the principal source of ECM proteins. Transforming growth factor (TGF)-β1 is the most potent driver of this process. NOX4 acts downstream of TGF-β1, through a ROS-induced Smad-independent mechanism involving mitogen-activated protein kinase (MAPK) pathways (i.e., ERK1/2, p38, JNK), thereby reinforcing the myofibroblast phenotype in a feed-forward loop [5,10,11,12]. TGF-β1 activates NOX4 primarily by enhancing its transcription via canonical SMAD2/3 signaling, which binds to regulatory elements within the NOX4 promoter and increases gene expression [10,11]. In parallel [10], TGF-β1 can further potentiate NOX4 activity through non-canonical pathways, including MAPK (ERK, JNK, p38) [10] and PI3K/AKT signaling [10], which promote NOX4 stabilization, intracellular trafficking, and mitochondrial localization [10]. Through these combined mechanisms, TGF-β1 establishes a redox-dependent feed-forward loop whereby increased NOX4-derived ROS amplify profibrotic signaling and sustain hepatic stellate cell activation.

Hepatocyte injury and apoptosis further propagate fibrosis. TGF-β1 upregulates mitochondrial NOX4, causing localized H_2_O_2_ production. This ROS surge triggers mitochondrial dysfunction, including loss of transmembrane potential, cytochrome c release, and caspase-3 activation, ultimately promoting hepatocyte death and sustaining fibrogenic signaling [10,13].

Hepatic macrophages, including resident Kupffer cells and recruited monocytes, orchestrate both inflammation and fibrosis resolution. NOX-derived ROS influence redox-sensitive transcription factors, such as NF-κB, affecting M1/M2 polarization [14]. Evidence suggests that NOX4’s effect on macrophages is context-dependent, at times promoting M2-like profibrotic phenotypes or, paradoxically, exacerbating fibrosis depending on the injury model [15,16]. Therefore, the TGF-β1–NOX4–ROS axis represents a key driver of HSC activation, promoting cytoskeletal remodeling [6], α-SMA expression, and extracellular matrix deposition through sustained redox-dependent signaling [7]. In parallel, NOX-derived ROS modulate macrophage function by influencing NF-κB activation and shaping the balance between M1 and M2 polarization, thereby amplifying the pro-fibrotic microenvironment [14].

**Figure 1:**
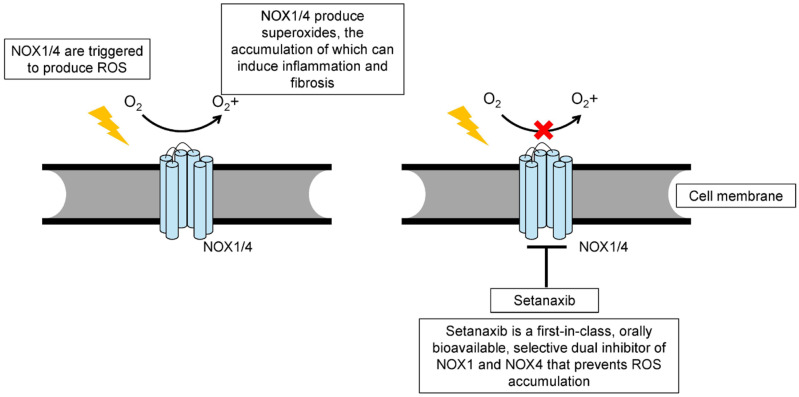
Schematic figure illustrating NOX1/4 effects and setanaxib mechanisms.

In this context, inhibition of NOX1 and NOX4 by setanaxib (Figure 1) may help rebalance the hepatic microenvironment away from chronic inflammation and fibrosis.

## 4. Preclinical Evidence for Setanaxib’s Antifibrotic Efficacy

The therapeutic potential of setanaxib (formerly GKT137831) has been robustly demonstrated in multiple, mechanistically distinct animal models of liver fibrosis (Table 2). In the bile duct ligation (BDL) model, which mimics cholestatic liver injury as seen in PBC, setanaxib treatment reduced HSC activation, hepatocyte apoptosis, and collagen deposition, improving liver architecture [17]. Similarly, in carbon tetrachloride (CCl_4_)-induced fibrosis, setanaxib suppressed α-smooth muscle actin (α-SMA) and collagen type I expression, normalized serum aminotransferases, and restored hepatic histology [7]. Mechanistically, these effects stem from interruption of the angiotensin II-Rac1-NOX1/4 signaling axis, dampening both inflammatory and chronic fibrogenic signaling. Complementary evidence comes from Studies in NOX4-deficient mice support these findings, showing enhanced liver regeneration after partial hepatectomy, suggesting that selective NOX4 inhibition may both prevent fibrosis and promote repair [18]. Nevertheless, NOX4 may have a dual role: in certain contexts, its deletion increased HCC risk in fibrotic livers, highlighting the need for careful patient selection [16]. This finding suggests a biphasic or context-dependent role for NOX4: it may act as a pro-fibrotic mediator during early and intermediate stages of disease but exert tumor-suppressive effects in the cirrhotic liver. These observations highlight the importance of careful patient selection and long-term safety monitoring in the clinical development of NOX4 inhibitors such as setanaxib.

## 5. Clinical Studies

Setanaxib exhibits favorable oral bioavailability and pharmacokinetic properties compatible with chronic administration [21]. In patients with PBC, treatment with setanaxib led to reductions in serum biomarkers of fibrogenesis, including procollagen type III N-terminal peptide (P3NP), providing direct evidence of target engagement and proof-of-mechanism in human disease [21]. However, P3NP reductions indicate modulation of ECM turnover but are imperfect measures of true fibrosis regression. Levels may fluctuate with inflammation, cholestasis or metabolic factors and do not necessarily reflect biopsy-proven improvement [21].

A Phase 2 randomized, double-blind trial (NCT03226067) evaluated setanaxib in 111 PBC patients with inadequate response to ursodeoxycholic acid (UDCA). Patients received placebo or setanaxib (400 mg once or twice daily) for 24 weeks. Although the primary composite biochemical endpoint was not met, secondary analyses revealed meaningful reductions in ALP and trends toward improved liver stiffness [20]. Notably, patients receiving 400 mg twice daily reported significant improvements in fatigue—a debilitating symptom in PBC—demonstrating a patient-centered benefit not consistently observed with other therapies [20]. Setanaxib was well tolerated, with mild-to-moderate gastrointestinal events and headache as the most common adverse effects [21]. Most adverse events were mild to moderate in severity, with gastrointestinal symptoms and headache being the most common, and their frequency comparable between treatment and placebo groups. Importantly, no dose-limiting toxicities, treatment-related serious adverse events, or cases of drug-induced liver injury were observed [20,21]. Furthermore, the selectivity of setanaxib for NOX1/4 over NOX2—the isoform essential for phagocytic host defense—was supported by the absence of increased infection risk [7]. It is important to underline that the primary composite biochemical endpoint of the Phase 2 trial was not met. This underscores possible limitations such as short study duration [22], heterogeneity of PBC, and restricted sensitivity of biochemical surrogates [22]. Secondary improvements (e.g., ALP reduction, trends in stiffness) should therefore be interpreted cautiously [20]. Moreover, although fatigue improvement is clinically relevant [23], it relies on subjective scales and requires cautious interpretation [24]. Another possibility related to the failure to meet the endpoints lies in potential dosing issues, which may not have ensured an optimal drug level [21]. Further clinical development of setanaxib is ongoing, with planned studies aiming to clarify its long-term efficacy, optimal dosing, and potential application in broader fibrotic liver diseases. These forthcoming trials are expected to better define the clinical relevance of NOX1/4 inhibition [19,21,25].

## 6. Discussion

### 6.1. Clinical Implications

The development of setanaxib from bench to bedside validates NOX1/4 inhibition as a mechanistically distinct and viable antifibrotic strategy. The accumulating body of evidence—including molecular studies, robust preclinical efficacy, and emerging clinical data—is compelling. Although the Phase 2 trial in PBC did not achieve its primary biochemical endpoint, the observed improvement in fatigue, coupled with a favorable safety profile and positive trends in liver stiffness, provides a strong rationale for continued clinical development.

Setanaxib’s mechanism—directly targeting the enzymatic source of pathological ROS in HSCs and hepatocytes—addresses a fundamental driver of fibrogenesis. Its selectivity for NOX1/4 spares the essential immune functions of NOX2, offering a significant safety advantage.

Although the precise antifibrotic mechanisms of NOX1/4 inhibition are not yet fully elucidated, several converging pathways provide a biologically plausible framework for its therapeutic potential. By dampening the TGF-β1–NOX4–ROS axis, setanaxib may reduce HSC activation [26] and extracellular matrix deposition [27], while the attenuation of NOX-derived ROS can modulate redox-sensitive transcriptional programs such as NF-κB and influence macrophage polarization away from profibrotic M2 phenotypes [28]. These interconnected redox-dependent processes offer a mechanistic rationale for the potential clinical efficacy of NOX1/4 inhibition, even as the full spectrum of molecular effects continues to be clarified [29].

Setanaxib’s mechanism complements other liver-directed therapies. FXR agonists, such as obeticholic acid (OCA), are approved for PBC and have been extensively studied in MASLD. FXR activation modulates bile acid metabolism, reduces hepatic lipogenesis, and exerts anti-inflammatory effects [30,31]. While FXR agonists have shown fibrosis improvement in some patients, their use is limited by side effects, such as pruritus and concerns regarding hepatic decompensation [32]. Setanaxib offers a complementary mechanism by directly targeting fibrogenic signaling in HSCs, and its fatigue-reducing effect is a unique differentiator. Therefore, combination therapy with an FXR agonist is a rational future approach. Similarly, glucagon-like peptide-1 (GLP-1) receptor agonists and multi-target incretins, such as semaglutide and tirzepatide, have demonstrated potent effects in MASLD by inducing weight loss and improving systemic metabolic health, thereby reducing the “fuel” for hepatic inflammation and steatosis [20,25,33,34]. However, their direct antifibrotic effects remain less well established. A combination of a metabolic agent like a GLP-1 agonist with a direct antifibrotic such as setanaxib could be highly synergistic, simultaneously targeting the metabolic “engine” of the disease and the fibrotic “machinery” of the liver [34].

### 6.2. Limitations

Several critical questions remain. The discrepancy between robust preclinical effects and modest biochemical outcomes in humans warrants further investigation, potentially involving dose optimization, patient selection, and refined endpoints [35]. The dual role of NOX4 in fibrosis and cancer underscores the importance of identifying candidates to such a treatment. Indeed, the demonstration that NOX4 deficiency may promote HCC in cirrhotic livers suggests that long-term, complete inhibition of NOX4 in patients with advanced cirrhosis may carry risks. This indicates that the optimal therapeutic window for NOX inhibition may lie in patients with moderate, rather than end-stage, fibrosis [16]. Isoform-specific inhibitors (NOX1-only or NOX4-only) could further clarify the individual contributions of each isoform and enable tailored therapy. Mechanistically, several hypotheses may explain why NOX4 loss predisposes to HCC in cirrhotic livers [16]. NOX4 contributes to redox homeostasis by generating controlled levels of hydrogen peroxide, which promotes apoptosis of damaged hepatocytes and limit uncontrolled proliferation [36]. In experimental models, NOX4 deletion reduces these tumor-suppressive checkpoints, enhances survival of genomically unstable hepatocytes [36], and favors a shift toward protumorigenic M2 macrophage polarization [16]. Thus, while NOX4 is clearly profibrotic in earlier disease stages, its absence in a cirrhotic microenvironment may remove an important brake on malignant transformation [36]. These observations suggest that NOX4 inhibition should be used cautiously in patients with advanced cirrhosis, and that the safest therapeutic window for dual NOX1/4 inhibition is likely before the development of late-stage fibrotic remodeling.

This lack of specificity is a scientific limitation in the drug’s mechanistic understanding [36]. Alternative strategies, such as antisense oligonucleotides, may offer even greater specificity [25].

### 6.3. Future Directions

Artificial intelligence (AI) offers opportunities to enhance clinical development. Multi-omic and imaging data could identify patients with high NOX activity who are most likely to respond to setanaxib. Quantitative digital pathology and radiology may serve as non-invasive surrogate endpoints, reducing reliance on liver biopsy. Explainable AI models could predict disease progression and optimize intervention timing [37,38,39].

## 7. Conclusions

Setanaxib, the first selective NOX1/4 inhibitor in clinical development, represents a shift from non-specific antioxidants to targeted redox modulation. Its mechanistic specificity, safety profile, and unique fatigue-reducing effect position it as a promising therapeutic option. By directly targeting ROS production in HSCs and hepatocytes, hepatic fibrosis may be reversible and molecularly reprogrammable. Future integration with metabolic therapies and AI-guided precision medicine may further enhance its clinical impact, marking a new era in antifibrotic hepatology. Overall, although targeted redox modulation represents a promising advance over non-specific antioxidant strategies [40], important challenges remain, including the potential oncogenic concerns linked to NOX4 inhibition [41], and the need for greater isoform selectivity [42] and patient stratification to fully realize the therapeutic potential of this approach in fibrotic disease [20].

## Figures and Tables

**Table 1:** Comparative Analysis of NOX1 and NOX4 in Hepatic Pathophysiology.

Feature	NOX1	NOX4
Primary Location	Plasma membrane	Mitochondria and other intracellular membranes
Activity Regulation	Inducible; requires assembly with cytosolic subunits (e.g., Rac1) for activation	Constitutively active; does not require inducible subunit assembly
Primary ROS Product	Superoxide (O_2_^−^)	Hydrogen Peroxide (H_2_O_2_)
Signaling Function	Drives rapid, acute inflammatory responses and cell proliferation	Acts as a more stable “second messenger” for intracellular redox signaling
Role in Fibrosis	Implicated in the initial inflammatory response and HSC proliferation	Critical for the sustained activation of HSCs into myofibroblasts and maintaining the chronic fibrogenic state

**Table 2:** Summary of Translational Evidence of Setanaxib.

Aspect	Key Findings and Evidence	Reference
Mechanism of action	Selective dual NOX1/4 inhibitor	[7]
	Attenuates HSC activation	[6]
	Modulates TGF-β signaling	[5]
	Reduces ECM deposition	[19]
	Regulates hepatic macrophage polarization.	[19]
Preclinical Efficacy (Animal Models)	In the BDL model (mimics PBC), setanaxib reduced HSC activation, hepatocyte apoptosis, and collagen deposition.	[17]
	In CCl_4_ model, it suppressed α-SMA and collagen type I expression.	[7]
Clinical Trial Evidence (Phase 2 in PBC)	Primary Endpoint: The primary composite biochemical endpoint was not met.	[20]
	Positive Outcomes: Meaningful reductions in ALP and trends toward improved liver stiffness were observed.	[20]
	Patient-Reported Outcome: Showed a significant improvement in fatigue.	[20]
Safety & Tolerability	Generally well-tolerated, with most adverse events being mild-to-moderate (e.g., GI events, headache).	[21]
	Key Safety Advantage: Spares NOX2 (essential for host defense), resulting in no observed increase in infection risk.	[7]
Key Nuance & Future Consideration	While NOX4 is profibrotic, its deletion in some models increased HCC risk in cirrhotic livers, which suggests the optimal therapeutic window for setanaxib may be in moderate, rather than end-stage, fibrosis.	[16]

## Data Availability

No new data were created or analyzed in this study. Data sharing is not applicable to this article.
